# Network modeling of the impact of social support and illness acceptance on disease activity in Crohn’s disease patients: a cross-sectional study

**DOI:** 10.3389/fpsyg.2025.1700857

**Published:** 2026-01-12

**Authors:** Jinli Bu, Jinghan Liu, Xiao Han, Zhen Wang, Junwei Ye, Meihao Wei

**Affiliations:** Nursing Department, Sir Run Run Shaw Hospital, Zhejiang University School of Medicine, Hangzhou, China

**Keywords:** Crohn’s disease, disease activity, illness acceptance, network analysis, social support

## Abstract

**Background:**

Crohn’s disease (CD) is a chronic inflammatory disorder marked by unpredictable disease activity and complex interactions between biological and psychosocial factors. Although social support and illness acceptance are known to influence patient outcomes, their combined impact on disease activity has not been systematically investigated.

**Objective:**

This study aimed to elucidate the interrelationships between social support, illness acceptance, and disease activity in patients with Crohn’s disease using network analysis.

**Methods:**

In a cross-sectional design, 276 CD patients completed standardized assessments, including the Multidimensional Scale of Perceived Social Support (MSPSS), the Acceptance of Illness Scale (AIS), and the Crohn’s Disease Activity Index (CDAI). A regularized partial correlation network was estimated using the graphical LASSO algorithm, with centrality and bridge strength metrics computed to identify influential variables. Subgroup analyses by marital status were conducted.

**Results:**

The network revealed key nodes with high centrality, including lack of self-worth (AIS7), feeling unneeded (AIS3), and lack of self-sufficiency (AIS6), along with perceived support from significant others. Bridge centrality analysis identified AIS7, family support, and overall disease activity as key bridging nodes connecting psychosocial and clinical domains. Subgroup analysis revealed distinct patterns: in single patients, family support and self-worth were directly linked to disease activity; in married patients, friend support and autonomy emerged as central, with no direct psychosocial-clinical links. Network structures did not significantly differ in global strength, but key node positions varied by marital status.

**Conclusion:**

These findings highlight self-worth, autonomy, and perceived social support as central psychosocial factors influencing disease activity in CD. These pathways differ by relationship status, underscoring the need for personalized psychosocial care strategies tailored to marital context. Longitudinal research is warranted to validate these dynamic relationships.

## 1 Introduction

Crohn’s disease (CD) is a chronic, progressive, and relapsing gastrointestinal inflammatory disease that affects the full thickness of the bowel wall (Dahlhamer et al., 2016; Sofia et al., 2020). It is estimated that the prevalence of CD in the United States is approximately 1.3%, and its global prevalence, including in Asia, continues to rise annually (Dahlhamer et al., 2016). This disease is often accompanied by complications such as intestinal strictures, fistulas, perforations, obstructions, abscesses, or perianal disease, significantly affecting the quality of life of both patients and their families (Cheifetz, 2013). Since the pathogenesis of CD remains unclear, the primary treatment strategy currently employed is pharmacological intervention aimed at slowing disease progression to reduce the need for surgery.

CD requires lifelong treatment. Reduced social support, along with anxiety and depression stemming from poor illness acceptance, may undermine patients’ confidence in managing their condition (Horrigan et al., 2023; Tzanetakos et al., 2025). This loss of confidence can result in poor treatment adherence, delayed healthcare-seeking behavior, and ultimately, worsened disease activity (Huang et al., 2023; van den Brink et al., 2018; Xiong et al., 2022). Moreover, female patients with inflammatory bowel disease (IBD) are more likely than males to experience psychological distress (Barberio et al., 2021; Huang et al., 2023). However, these studies mostly consider direct factors influencing CD activity, such as anxiety and depression, with limited attention to the underlying causes of these psychological factors.

Social support is derived from various sources, including family members, friends, and other networks (Kamp et al., 2019). Illness acceptance may be another dimension influencing CD activity. Interestingly, there has been little research exploring the interrelationship between social support, illness acceptance, and disease activity in CD patients.

Network analysis, as a relatively new statistical method, is used to model the complex interactions between multiple variables. It allows for the visualization of specific relationships between variables within the network and identifies key variables within the analysis. Exploring the network of social support, illness acceptance, and disease activity in CD can help elucidate which variables are most influential and may offer new approaches for developing or optimizing interventions to reduce disease activity. Additionally, age-related differences may lead to variations in the reported variables (Chen et al., 2023; Li et al., 2023).

Therefore, the first objective of this study is to apply network analysis to explore the complex interactions between social support, psychological perceptions, and disease activity in CD patients. By evaluating the network relationships between specific variables, we aim to deepen the understanding of how social support and psychological perceptions affect disease activity through particular variables, and to identify core social support and psychological perception variables in CD patients. The second objective is to examine whether these core variables differ by gender. A better understanding of how gender differences influence the relationships between core variables may provide new insights into developing gender-specific interventions targeting social support and psychological factors for disease acceptance.

## 2 Materials and methods

### 2.1 Study design and setting

Participants diagnosed with CD at a tertiary hospital in Zhejiang between December 2024 and May 2025 were recruited for this study. Participants were invited to join the study and complete a questionnaire during their hospitalization. Eligible participants were required to meet the following inclusion and exclusion criteria. Inclusion criteria: (1). Diagnosis of CD according to the 2021 “Consensus on the Diagnosis and Treatment of IBD in China.” (2). Age between 18 and 45 years; (3). Ability to understand and complete the questionnaire. Exclusion criteria: (1). Co-existing severe psychiatric disorders; (2). Experiencing a major traumatic event within the past 3 months. Participants were recruited during a hospitalization episode, capturing a clinical state often characterized by increased disease activity or treatment escalation. The study was approved by the Medical Ethics Committee of the tertiary hospital in Zhejiang (Approval No. 2024-2,610-01).

### 2.2 Measurements

#### 2.2.1 Demographic information

The following variables were collected from the participants: age, gender, height, weight, education level, monthly income, disease duration, whether they received biologic biosimilar therapies, whether they received immunosuppressive treatments, whether they underwent surgical treatment, and the number of chronic conditions such as diabetes and hypertension.

#### 2.2.2 Illness acceptance

Illness acceptance was assessed using the Illness Acceptance Scale (AIS). The original AIS was developed by Felton and Revenson (1984) and has been widely used and validated in Chinese populations, including patients with chronic diseases such as diabetes and cancer (Chabowski et al., 2017; Felton and Revenson, 1984; Gao et al., 2025; Li et al., 2025a; Li et al., 2025b; Liu X. et al., 2025; Ma et al., 2025). The AIS contains eight items (AIS1-8) designed to measure the degree of patient adaptation to their illness. These items assess: (1) how well the individual adapts to the limitations caused by the illness, (2) whether they are able to engage in their favorite activities, (3) whether they perceive others as uncomfortable with their illness, (4) whether they feel unnecessary, (5) a sense of worthlessness, (6) lack of self-sufficiency, (7) reliance on others, and (8) whether they feel they are a burden to their family and friends. Each item is rated on a five-point Likert scale ranging from 1 (strongly agree) to 5 (strongly disagree), where a score of 1 indicates poor adaptation to the illness and a score of 5 indicates complete acceptance. The total score ranges from 8 to 40, with higher scores indicating better acceptance of the illness and fewer associated negative emotions and psychological distress. The Cronbach’s alpha coefficient for the Polish version of the AIS was 0.82, indicating good internal consistency.

#### 2.2.3 Multidimensional scale of perceived social support (MSPSS)

Social support was measured using the Multidimensional Scale of Perceived Social Support (MSPSS), developed by Calderón et al. (2021). The MSPSS has been extensively applied in various Chinese patient groups, demonstrating robust psychometric properties (Hu et al., 2025; Kang et al., 2024; Liu S. et al., 2025; Zhao et al., 2022). This scale aims to assess an individual’s perception of the adequacy of social support from three sources: family (items 3, 4, 8, and 11), friends (items 6, 7, 9, and 12), and significant others (items 1, 2, 5, and 10). Respondents rate each item using a seven-point Likert scale, where 1 represents “strongly disagree” and 7 represents “strongly agree.” The total score ranges from 12 to 84, with higher scores indicating stronger perceived social support. The MSPSS demonstrates good internal consistency, with a Cronbach’s alpha of 0.95, indicating high reliability (Zimet et al., 1990).

#### 2.2.4 The simplified CD activity index (CDAI)

The Simplified CD Activity Index (CDAI), also known as the Harvey–Bradshaw Index (HBI), is commonly used to assess clinical activity in CD patients. The CDAI is a well-established, clinician-friendly instrument that has been routinely employed in both international and Chinese clinical research and practice for monitoring CD activity, and its validity has been supported by correlations with endoscopic and radiographic findings (Ding et al., 2022; Lin et al., 2018; Wu et al., 2020). The index includes five components: overall health, abdominal pain, diarrhea, abdominal mass, and associated complications. It is based on the patient’s symptoms over the past 7 days. Scores from 0 to 4 indicate disease remission, scores from 5 to 8 indicate moderate disease activity, and scores of 9 or higher indicate severe disease activity (CD).

### 2.3 Statistical analysis

For continuous variables that follow a normal distribution, means and standard deviations (SD) were used. Otherwise, medians and interquartile ranges (IQR) were reported. Categorical variables were described using frequencies and percentages. The differences in baseline characteristics between groups were compared using chi-square tests (χ2), analysis of variance (ANOVA), and the Kruskal-Wallis rank sum test. The analysis was also stratified by gender. All statistical analyses were performed using R software (version 4.4.3), and a *p*-value of <0.05 was considered statistically significant.

#### 2.3.1 Network estimation and visualization

Graphical Least Absolute Shrinkage and Selection Operator (graphical LASSO, glasso) algorithm was used to estimate the partial correlation network between social support, illness acceptance, and disease activity in CD patients. Visualization was performed using the qgraph package in R. In the network model, nodes represent the variables included in the analysis, including dimensions of social support, illness acceptance, and disease activity indicators. Edges indicate the conditional dependence between two nodes after controlling for all other variables. To avoid spurious correlations and enhance the sparsity of the model, parameter tuning was performed using the Extended Bayesian Information Criterion (EBIC; Chen and Chen, 2008), shrinking weaker and unstable edges to zero, thereby resulting in a more robust and interpretable network structure. The network graph was visualized using the Fruchterman–Reingold layout algorithm (Fruchterman and Reingold, 1991). This algorithm positions strongly connected nodes close together, while weakly connected nodes are placed further apart. The thickness and saturation of the edges reflect the strength of the partial correlation coefficient, with positive correlations shown in blue and negative correlations in red. To account for potential gender differences, separate network models were constructed for male and female groups to explore the gender-specific influence of social support and illness acceptance on disease activity.

#### 2.3.2 Network inference and stability

To explore the importance of social support and illness acceptance in the CD activity network and gender differences, node centrality analysis was employed to assess the strength of node connections with other nodes in the network, including node strength (Strength), closeness (Closeness), and betweenness (Betweenness) metrics. To consider the influence of both positive and negative edges, we also calculated the expected influence (EI), a metric that retains both edge weights’ signs (Robinaugh et al., 2016).

All centrality metrics were calculated using the qgraph package in R and standardized using Z-scores for comparison. To verify the stability of the results, bootstrapping analysis (nboots = 2000) was conducted using the bootnet package, estimating the 95% confidence intervals for edge weights and calculating the node strength stability coefficient (CS-coefficient). Following the guidelines of Epskamp et al. (2018) and Zhou et al. (2024)., a CS coefficient greater than 0.25 is considered interpretable, and a CS coefficient greater than 0.50 indicates good stability.

In addition, to further explore the role of marital status, a subgroup network analysis was conducted comparing patients with and without partners. Two separate regularized partial correlation networks were estimated for each group, and network invariance was assessed using the Network Comparison Test (NCT).

## 3 Results

### 3.1 Sample characteristics

Table 1 presents the demographic and clinical characteristics of the study sample. A total of 276 patients with CD were included, with a median age of 28 years (IQR: 23.0–34.0). The majority of participants were male (79.0%, *n* = 218). Most patients had received biologic biosimilar therapies (90.9%) and nearly half had undergone surgical treatment (49.6%). Notably, female participants reported significantly higher levels of perceived social support from friends and had lower BMI values compared to males (*p* < 0.05).

**Table 1:** Demographics of participants with Crohn’s disease based on gender.

Variable	Total (*n* = 276)	Male (*n* = 218)	Female (*n* = 58)	*p* value
Age, years (mean ± SD)	28.0 [23.0–34.0]	28.0 [23.2–34.0]	27.5 [23.0–34.0]	0.844
BMI, kg/m^2^ (mean ± SD)	21.5 [19.2–24.5]	21.9 [19.5–25.2]	20.4 [18.0–22.6]	0.001
Education level				1.000
High school or below	47 (17.0%)	37 (17.0%)	10 (17.2%)	
University or above	229 (83.0%)	181 (83.0%)	48 (82.8%)	
Monthly income, RMB, *n* (%)				0.304
<5,000	40 (14.5%)	28 (12.8%)	12 (20.7%)	
5,000–10,000	123 (44.6%)	98 (45.0%)	25 (43.1%)	
>10,000	113 (40.9%)	92 (42.2%)	21 (36.2%)	
Disease duration, years, *n* (%)				0.605
<5	182 (65.9%)	141 (64.7%)	41 (70.7%)	
5–10	66 (23.9%)	55 (25.2%)	11 (19.0%)	
>10	28 (10.1%)	22 (10.1%)	6 (10.3%)	
Biologic biosimilar therapies, *n* (%)				1.000
Yes	251 (90.9%)	198 (90.8%)	53 (91.4%)	
No	25 (9.06%)	20 (9.17%)	5 (8.62%)	
Immunosuppressants, *n* (%)				0.808
Yes	61 (22.1%)	47 (21.6%)	14 (24.1%)	
No	215 (77.9%)	171 (78.4%)	44 (75.9%)	
Surgical treatment for IBD, *n* (%)				0.499
Yes	137 (49.6%)	111 (50.9%)	26 (44.8%)	
No	139 (50.4%)	107 (49.1%)	32 (55.2%)	
The number of chronic diseases, *n* (%)				0.235
0	161 (58.3%)	122 (56.0%)	39 (67.2%)	
1	101 (36.6%)	83 (38.1%)	18 (31.0%)	
≥2	14 (5.07%)	13 (5.96%)	1 (1.72%)	
CDAI general condition, *n* (%)				0.576
2	206 (74.6%)	159 (72.9%)	47 (81.0%)	
3	60 (21.7%)	51 (23.4%)	9 (15.5%)	
4	5 (1.81%)	4 (1.83%)	1 (1.72%)	
5	5 (1.81%)	4 (1.83%)	1 (1.72%)	
CDAI abdominal pain, *n* (%)				0.734
2	162 (59.1%)	130 (60.2%)	32 (55.2%)	
3	99 (36.1%)	76 (35.2%)	23 (39.7%)	
4	13 (4.74%)	10 (4.63%)	3 (5.17%)	
CDAI diarrhea, *n* (%)	1.00 [1.00–2.00]	1.00 [1.00–2.00]	1.00 [1.00–2.00]	0.845
CDAI abdominal mass, *n* (%)				1.000
2	251 (91.3%)	198 (91.2%)	53 (91.4%)	
3	15 (5.45%)	12 (5.53%)	3 (5.17%)	
4	9 (3.27%)	7 (3.23%)	2 (3.45%)	
CDAI comorbidities, *n* (%)	1.00 [1.00–2.00]	1.00 [1.00–2.00]	1.00 [1.00–2.00]	0.773
CDAI scores, *n* (%)	5.00 [3.00–6.00]	5.00 [3.00–6.00]	5.00 [3.00–7.00]	0.649
Family Support, *n* (%)	23.0 [18.0–26.0]	22.5 [18.0–26.0]	24.0 [20.0–26.0]	0.183
Friend support, *n* (%)	20.0 [16.0–24.0]	20.0 [16.0–24.0]	22.0 [18.2–24.0]	0.026
Other support, *n* (%)	20.0 [16.0–24.0]	20.0 [16.0–24.0]	21.0 [17.0–24.0]	0.506
Comprehensibility, *n* (%)	20.0 [15.0–23.0]	20.0 [15.2–23.0]	20.0 [15.0–23.0]	0.857
Manageability, *n* (%)	18.0 [12.0–21.0]	18.0 [12.0–21.0]	17.0 [12.0–20.0]	0.490
Meaningfulness, *n* (%)	17.0 [15.0–19.0]	17.0 [15.0–19.0]	17.0 [15.2–19.0]	0.853
Total social support, *n* (%)	62.0 [52.0–72.0]	62.0 [50.0–72.0]	64.5 [59.0–72.0]	0.168

The continuous variables were presented as median (interquartile range, IQR), and the categorical variables were shown as number and percentages. BMI, body mass index; SD, standard deviation; IBD, Inflammatory Bowel Disease; CDAI, the Crohn’s Disease Activity Index.

### 3.2 Network structure of social support, illness acceptance, and disease activity

**Figure 1:**
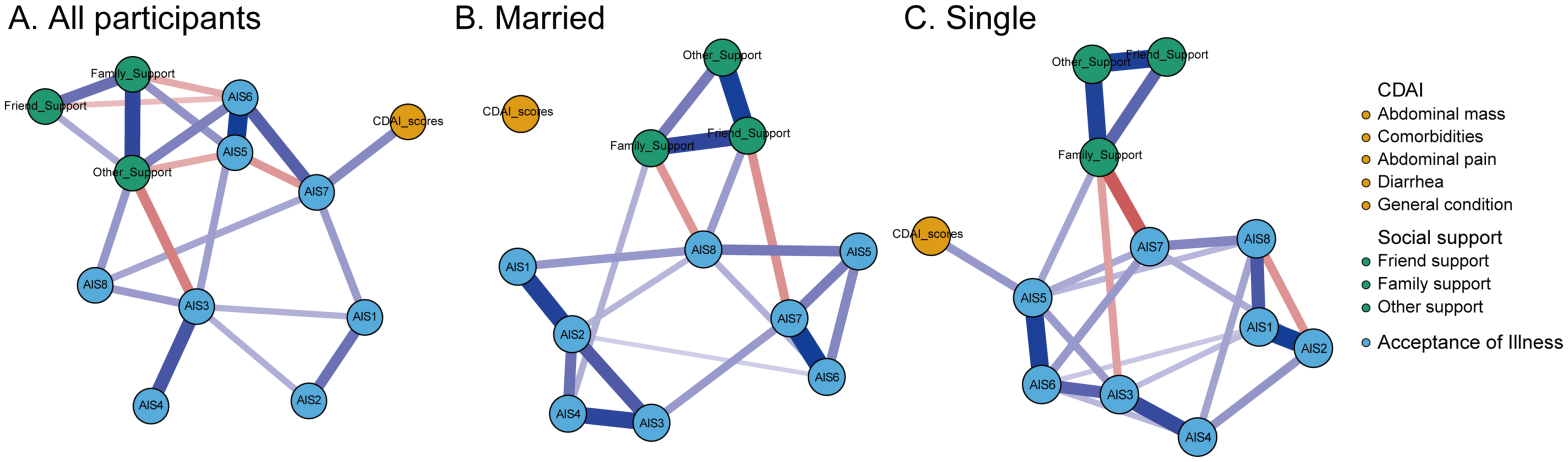
Regularized network analysis of the relationships between social support, disease acceptance, and disease activity in Crohn’s disease patients, stratified by marital status. The thickness of the lines between nodes represents the strength of their relationships, with blue indicating positive correlations and red indicating negative correlations. AIS1, adaptation to illness limitations; AIS2, ability to engage in favorite activities; AIS3, perception of others' discomfort with illness; AIS4, feeling unnecessary; AIS5, sense of worthlessness; AIS6, lack of self-sufficiency; AIS7, reliance on others; AIS8, feeling like a burden to family and friends; CDAI, the Crohn’s Disease Activity Index. **(A)** All particpiants, **(B)** Married, and **(C)** Single.

Figure 1 illustrates the estimated partial correlation network comprising illness acceptance (AIS items), social support (MSPSS subscales), and overall disease activity (CDAI total score). The network was estimated using the graphical LASSO algorithm with EBIC tuning to enhance sparsity and interpretability. In the visualization, nodes represent individual questionnaire items or scale dimensions, and edges represent regularized partial correlations between variables after conditioning on all others. Edge thickness and saturation reflect the strength of associations, with blue indicating positive correlations and red indicating negative ones.

The overall network displayed a moderate density, with several nodes showing strong interconnections, particularly within the domains of illness acceptance and social support. Crucially, overall disease activity (CDAI total score) was directly connected to multiple psychological and social variables, suggesting that psychosocial factors play an integral role in shaping disease outcomes.

To further investigate the influence of marital status, subgroup analyses were conducted separately for married (*n* = 106) and single (*n* = 170) participants. Distinct network configurations emerged across these two groups.

In the married subgroup, friend support emerged as the most central node in the network, demonstrating strong positive associations with family support, other support, and the illness acceptance item “feeling like a burden to family and friends” (AIS8). Conversely, friend support was negatively associated with “reliance on others” (AIS7), suggesting a nuanced relationship between perceived peer support and autonomy-related illness acceptance. Additionally, the item “ability to engage in favorite activities” (AIS2) also exhibited high centrality, showing broad positive associations with other illness acceptance dimensions and social support sources. However, in this subgroup, none of the psychosocial variables showed a direct connection with disease activity, suggesting a decoupling between subjective support and clinical status in partnered individuals.

In contrast, the single subgroup exhibited a different pattern. Family support was identified as the most central node, strongly linked to friend support, other support, and the illness acceptance item “sense of worthlessness” (AIS5). Importantly, family support exerted an indirect effect on disease activity, mediated through AIS5. The item sense of worthlessness also demonstrated high centrality and served as a key bridge node, showing a direct positive association with disease activity (CDAI total score). These findings suggest that among single patients, perceived family support and psychological vulnerability—particularly feelings of low self-worth—may constitute critical pathways influencing disease severity. Overall, these subgroup analyses reveal important differences in the psychosocial network architecture by marital status.

### 3.3 Centrality analysis

**Figure 2:**
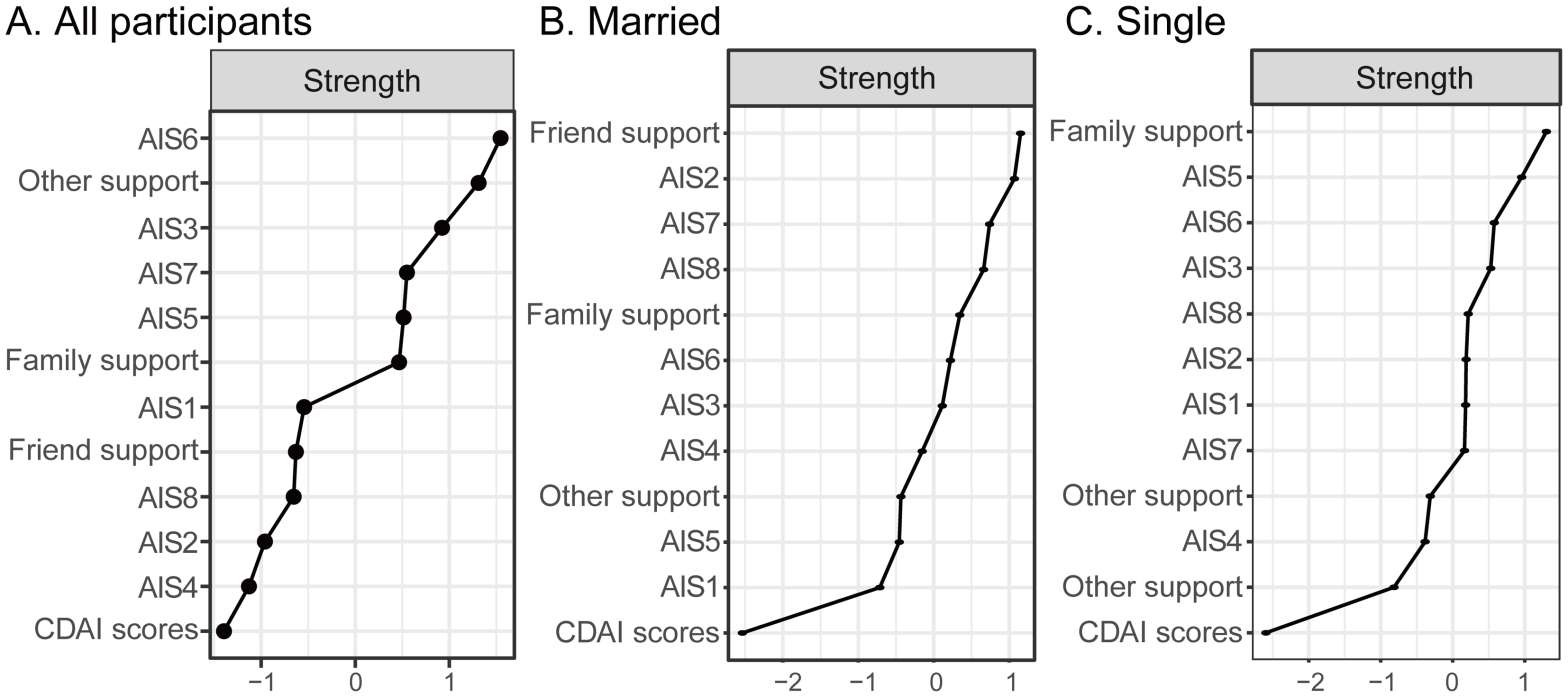
Standardized centrality indices (*z*-scores) for all nodes in Crohn’s disease patients, stratified by marital status. Higher standardized strength values indicate greater centrality within the network. AIS1, adaptation to illness limitations; AIS2, ability to engage in favorite activities; AIS3, perception of others’ discomfort with illness; AIS4, feeling unnecessary; AIS5, sense of worthlessness; AIS6, lack of self-sufficiency; AIS7, reliance on others; AIS8, feeling like a burden to family and friends; CDAI, the Crohn’s disease activity index. **(A)** All particpiants, **(B)** Married, and **(C)** Single.

Centrality indices (Figure 2) revealed that the most central nodes in the network — based on standardized node strength — were: AIS6 (lack of self-sufficiency), AIS3 (feeling unnecessary), AIS7 (loss of self-worth), Other support (support from significant others). These nodes exhibited the highest degree of connectedness, indicating their pivotal roles in the interplay between psychological adjustment and disease activity. In addition to the central nodes, the network analysis also revealed several weaker or non-significant edges. Specifically, items AIS2 and AIS4 were located at the periphery of the network, showing relatively weak connections with other variables. These weak associations suggest that these aspects of illness acceptance may have a less direct impact on disease activity or may be more influenced by other psychosocial factors. The weak edges highlight the complexity of the relationships between the psychosocial dimensions and disease activity, suggesting that not all aspects of illness acceptance exert the same degree of influence on disease outcomes.

In the married subgroup, the core variables were “friend support” and “ability to engage in favorite activities” (AIS2). The single subgroup presented a different pattern. “Family support” emerged as the most central node, highlighting the critical role of familial relationships in the psychosocial structure of individuals without partners. Importantly, “sense of worthlessness” (AIS5) also exhibited high centrality and was directly linked to disease activity (CDAI total score), suggesting a stronger psychosomatic interface in this group.

### 3.4 Bridge centrality analysis

**Figure 3:**
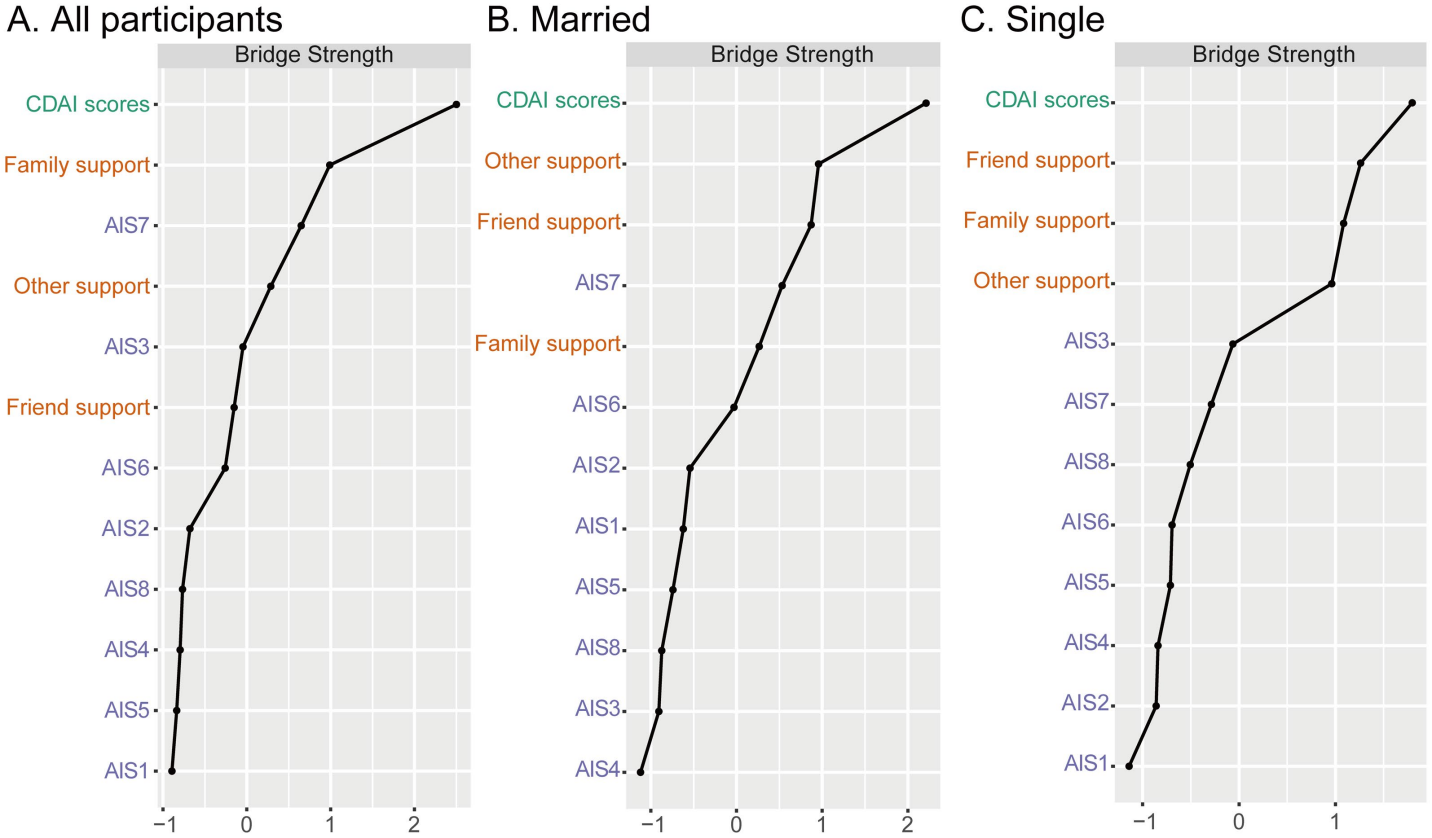
Standardized bridging strength centrality scores for nodes in the networks of social support, disease acceptance, and disease activity, stratified by marital status. Higher values indicate greater bridging centrality. AIS1, adaptation to illness limitations; AIS2, ability to engage in favorite activities; AIS3, perception of others’ discomfort with illness; AIS4, feeling unnecessary; AIS5, sense of worthlessness; AIS6, lack of self-sufficiency; AIS7, reliance on others; AIS8, feeling like a burden to family and friends; CDAI, the Crohn’s Disease Activity Index. **(A)** All particpiants, **(B)** Married, and **(C)** Single.

Bridge strength analysis (Figure 3) identified the key variables that act as bridges between the domains of illness acceptance, social support, and disease activity. The top bridging nodes included: AIS7 (loss of self-worth), Family support (MSPSS subscale), Overall disease activity score (CDAI total).

These findings suggest that the feeling of diminished self-worth and reduced family support may serve as critical psychological-somatic connectors that influence the exacerbation or alleviation of CD activity.

In the married subgroup, the most prominent bridging variables were Other support (support from significant others), Friend support, and AIS7 (loss of self-worth). In the single subgroup, Friend support, Family support, and CDAI total score emerged as the strongest bridging nodes. The presence of CDAI among the top bridge variables suggests a more direct psychosocial-clinical interface in this group. For single individuals, support from both friends and family—likely the primary sources of social contact—may have a more immediate bearing on their illness perception and disease management. The bridging role of Family support in this subgroup reinforces the critical compensatory role of familial networks in the absence of a partner.

### 3.5 Stability and accuracy of the network

**Figure 4:**
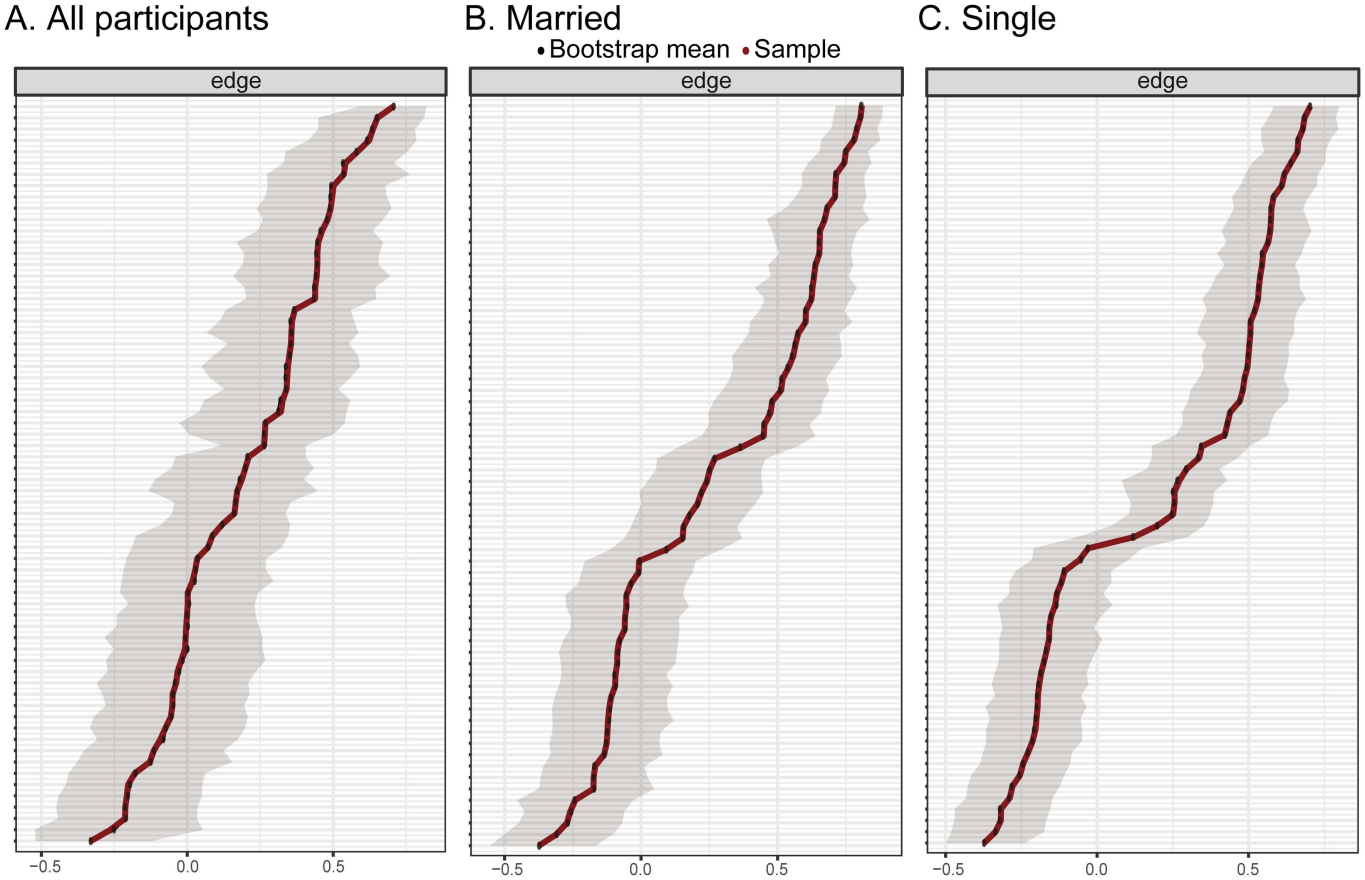
Stability analysis of centrality measures in the overall sample of Crohn’s disease patients. The *y*-axis represents the mean correlation coefficient between the centrality measures of the original sample and those of subsamples as the number of cases decreases. The line represents the mean, and the shaded area indicates the 95% confidence interval. **(A)** All particpiants, **(B)** Married, and **(C)** Single.

**Figure 5:**
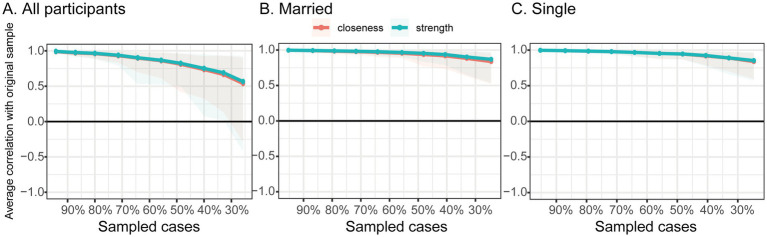
Bootstrapped 95% confidence intervals for the estimated edge weights in the network of social support, disease acceptance, and disease activity in the overall, married and single groups. **(A)** All particpiants, **(B)** Married, and **(C)** Single.

**Figure 6:**
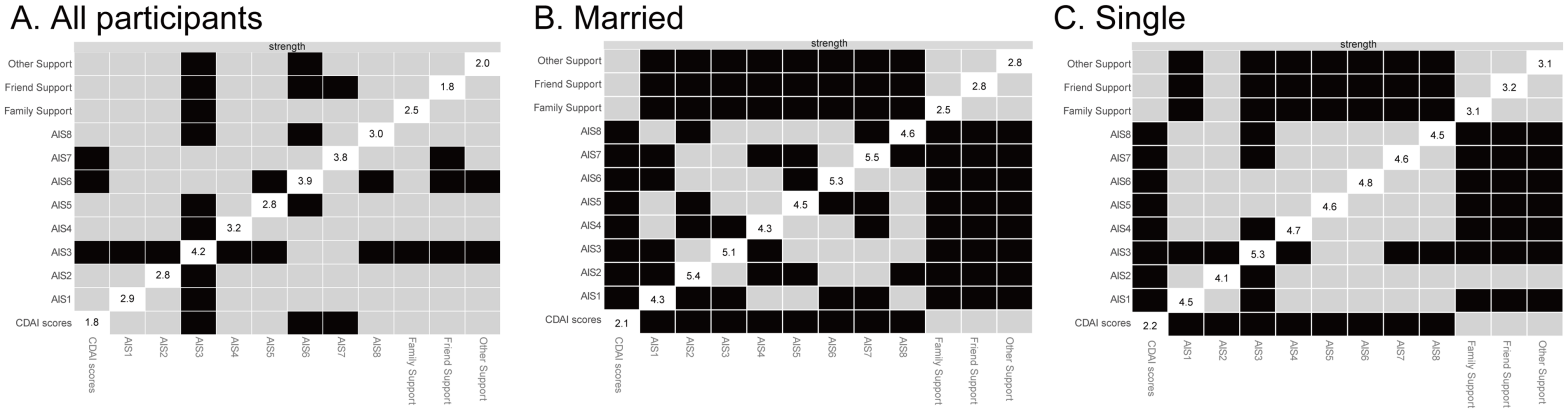
Bootstrapped difference tests (*α* = 0.05) for social support, disease acceptance, and disease activity in the overall, married and single groups. Gray boxes indicate no significant strength differences between nodes, while black boxes indicate significant strength differences. The strength values for each node are shown along the diagonal. AIS1, adaptation to illness limitations; AIS2, ability to engage in favorite activities; AIS3, perception of others’ discomfort with illness; AIS4, feeling unnecessary; AIS5, sense of worthlessness; AIS6, lack of self-sufficiency; AIS7, reliance on others; AIS8, feeling like a burden to family and friends; CDAI, the Crohn’s Disease Activity Index. **(A)** All particpiants, **(B)** Married, and **(C)** Single.

Bootstrapped confidence intervals for edge weights and the correlation stability (CS) coefficient were used to assess the robustness of the network (Figure 4). The CS-coefficient for node strength in the total sample was 0.35, which exceeds the minimum acceptable threshold of 0.25 and thus indicates interpretable, though moderate, stability of the centrality indices. However, it falls below the preferred threshold of 0.50, suggesting that the results should be interpreted with some caution. In addition, bootstrapped edge-weight CIs (Figures 5, 6) demonstrated relatively narrow intervals for most connections, supporting the accuracy and interpretability of the network structure.

To further explore the influence of marital status on network robustness, separate stability analyses were conducted for the married and single subgroups. In both subgroups, the CS coefficient for node strength reached 0.67, surpassing the preferred threshold of 0.50 and indicating a high level of centrality stability. This suggests that the network structures derived within each marital status subgroup are considerably more robust and interpretable than the total sample network. These findings provide additional confidence in the subgroup-specific network patterns, including the identification of distinct bridging nodes within the psychosocial and clinical domains.

### 3.6 Network comparison between married and single subgroups

To examine whether marital status influenced the network structure of perceived social support, illness acceptance, and disease activity, we conducted a NCT between the married and single subgroups. This analysis evaluated both network structure invariance and global strength invariance across the two groups.

Results indicated that there were no statistically significant differences in the overall network structure or global connectivity strength between the married and single groups (*p* > 0.05; Table 2). These findings suggest that the general configuration of associations among psychosocial and clinical variables is preserved across marital status, despite minor variations in the strength of specific edges.

**Table 2:** Comparison of global network features and structural characteristics across marital status groups.

Global strength and *p*-values	All participants (*n* = 276)	Married participants (*n* = 106)	Single participants (*n* = 170)	Network 1 vs. 2	Network 1 vs. 3	Network 2 vs. 3
Global strength	10.88	11.36	11.33			
Top 5 most central nodes (strength centrality)	AIS5 (1.17)	Friend support (1.38)	Family support (1.33)			
	AIS3 (1.13)	AIS2 (1.32)	AIS5 (1.23)			
	Family support (1.13)	AIS7 (1.22)	AIS6 (1.11)			
	AIS6 (1.03)	AIS8 (1.19)	AIS3 (1.10)			
	Other support (0.88)	Family support (1.08)	AIS8 (1.01)			
NCT results						
Omnibus test of network structure invariance p-value				0.95	1	0.742
Global strength invariance test p-value				0.564	0.257	0.208

NCT, network comparison test; Network 1, all participants; Network 2, married participants; Network 3, single participants.

## 4 Discussion

This study is the first to apply network analysis to explore the interrelationships among social support, illness acceptance, and disease activity in patients with CD, identifying key variables that play central roles in maintaining the structure of this network. Among the illness acceptance dimensions, AIS6 (perceived lack of self-sufficiency), AIS3 (feeling unneeded), and AIS7 (loss of self-worth), along with “other support” from the social support scale, were identified as the nodes with the highest centrality. Furthermore, total disease activity score, family support, and AIS7 also exhibited the highest bridge strength, acting as critical connectors among the three domains—disease activity, social support, and illness acceptance. These findings highlight the complex interdependence among psychosocial factors and disease activity, suggesting that feelings of worthlessness and self-insufficiency may contribute to heightened disease activity in CD. In addition, while the overall network structure showed invariance between married and single patients (as indicated by the Network Comparison Test), the centrality and bridging roles of specific nodes differed markedly. This suggests that the relative importance and functional pathways through which psychosocial factors influence disease activity are context-dependent, hinging on the individual’s primary relational structure.

Notably, AIS7 (loss of self-worth) and AIS3 (feeling unneeded) emerged as the most central psychological nodes, reflecting maladaptive cognitive patterns and diminished psychological resilience. The centrality of self-worth underlines its key influence on disease dynamics. Clinically, low perceived self-worth (AIS7) appears to be the most impactful symptom node, with strong connections to both illness acceptance and disease activity. Internalization of negative self-perception may lead to a cascade of maladaptive outcomes, consistent with prior evidence linking self-worth impairment to emotional dysregulation, reduced treatment adherence, and increased inflammatory response in IBD patients (Keefer et al., 2011; Opheim et al., 2020; Wu et al., 2022).

Emerging evidence has shown that decreased self-esteem may contribute to the physiological burden of chronic inflammatory conditions by activating neuroendocrine and immune pathways. For instance, Ghia et al. (2009). demonstrated that depression can reactivate quiescent colitis in a murine model of IBD by disrupting the cholinergic anti-inflammatory reflex via *α*7 nicotinic acetylcholine receptors on macrophages. This leads to increased secretion of proinflammatory cytokines, such as TNF-α and IL-6. Treatment with antidepressants prevented this reactivation, highlighting the role of psychological state in modulating inflammation through neuroimmune mechanisms in IBD. Similarly, Seohyun et al. found that low self-esteem was associated with lower medication adherence and increased healthcare utilization (Jeong et al., 2025).

In this study, AIS7 also functioned as a bridge node linking psychological dimensions with disease activity, suggesting that interventions targeting self-worth may have wide-ranging clinical benefits. Psychological interventions such as cognitive-behavioral therapy (CBT) have been shown to enhance self-worth and improve disease outcomes in IBD patients, indicating that AIS7 may serve as a strategic clinical target to reduce CD activity via both direct and indirect pathways (Wang et al., 2023).

Another key psychological node, AIS6 (lack of autonomy), reflects patients’ dependency and diminished self-management capabilities. In chronic disease models, learned helplessness has been repeatedly associated with poor prognosis (Borysenko, 1982). Shared decision-making and self-management education have demonstrated benefits in empowering IBD patients and reducing stress-related flare-ups (Chen, 2016). From a neuropsychological perspective, “learned helplessness” linked to AIS6 may be associated with dysregulation of the hypothalamic–pituitary–adrenal (HPA) axis, a key mechanism in CD pathophysiology (Liu et al., 2012). Interventions aimed at restoring self-efficacy, such as motivational interviewing or resilience training, may thus yield synergistic benefits on both emotional well-being and disease remission.

However, the marital status subgroup analysis adds a critical layer of nuance. In single patients, the direct bridge between sense of worthlessness (AIS5) and disease activity was stark, and family support influenced disease activity indirectly through this node. This paints a picture where, in the absence of a spousal partner, self-worth becomes the primary psychosomatic conduit, highly sensitive to the quality of familial support. Conversely, in married patients, self-worth (loss of self-worth, AIS7) retained centrality but its connection to disease activity was less direct, appearing more as a bridge within the psychosocial domain (linking to friend support). This implies that the marital relationship may buffer the direct physiological impact of low self-worth, possibly by providing an alternative source of validation and practical coping assistance, thereby “decoupling” negative self-perception from disease exacerbation.

Within the Multidimensional Scale of Perceived Social Support (MSPSS), family support and support from significant others were identified as either central or bridging nodes. These findings align with prior research showing that perceived social support is one of the strongest psychosocial predictors of health-related quality of life (HRQoL) in IBD (Chen et al., 2022). These social support components are crucial for buffering psychological distress and potentially mitigating disease activity. Patients with strong family or partner support often exhibit greater emotional resilience, higher adherence to treatment, and fewer clinical deteriorations (Huang et al., 2021). Beyond a protective effect, social support may also prevent internalization of stigma and negative affective states. Longitudinal studies such as that by Tinbete et al. found that higher family support was associated with lower relapse rates and better medication adherence in patients with chronic mental disorders (Samuel et al., 2022). Furthermore, intimate partner support has been linked to lower hospitalization rates and fewer complications in chronic diseases (Larsen et al., 2023). These findings suggest that enhancing family and intimate support networks could serve as an adjunctive therapeutic avenue. In clinical practice, this could involve implementing family psychoeducation or peer support models, particularly for newly diagnosed or relapsing patients.

Through network analysis, this study provides a systems-level perspective on the psychosocial architecture of CD, identifying high-centrality nodes that could serve as targets for precision interventions. Whereas traditional interventions have largely focused on anxiety and depression, our results suggest that upstream drivers such as diminished self-worth, loss of autonomy, and lack of close social support may be more central to disease activity. From a precision medicine perspective, patients exhibiting high-centrality traits such as low self-worth or poor social support may benefit from targeted psychological therapies (e.g., CBT, acceptance and commitment therapy, compassion-focused therapy), structured social support interventions (including caregiver involvement and peer groups), and training in autonomous disease self-management behaviors.

Additionally, the findings underscore the importance of routine psychosocial screening in clinical care. Incorporating brief instruments such as the MSPSS and AIS into outpatient settings may enable early identification of patients at elevated risk due to psychosocial vulnerability. Clinicians can then prioritize early intervention and refer patients to psychosocial services. Training IBD nurses and gastroenterologists in psychosocial assessment may further support early detection and the implementation of integrative care models.

Our findings are consistent with and extend previous work examining the role of psychosocial factors in Crohn’s disease activity. For instance, a 2024 study by de Dios-Duarte and colleagues demonstrated that CD patients undergoing flare-ups reported significantly higher stress levels, and that insufficient social support exacerbated these stress responses, contributing to increased flare-up incidence (de Dios-Duarte et al., 2024). Similar to their findings, our results highlight the protective role of social support in modulating disease activity. However, our study further contributes to this field by employing a network analytic approach, which enables a nuanced identification of how specific dimensions of illness acceptance (e.g., self-worth and autonomy) and types of perceived social support (e.g., family vs. friend) interact with disease activity.

Whereas the study by de Dios-Duarte et al. treated social support and stress as aggregate constructs, our network model delineates node-specific pathways, revealing that perceived self-worth (AIS7) and family support function as bridge nodes between psychological and somatic dimensions. This suggests potential mechanistic targets for psychosocial interventions. Moreover, the stratified network structure by marital status observed in our study provides evidence that the psychosocial architecture of disease vulnerability is context-dependent, a feature not addressed in previous cross-sectional studies. Together, these complementary findings reinforce the importance of early identification of psychosocial vulnerabilities and tailoring psychosocial interventions according to individual support structures and illness appraisals.

Several limitations should be acknowledged. First, the cross-sectional design precludes causal inferences; longitudinal studies are needed to confirm temporal dynamics. Second, although validated scales were used, self-reported data may be subject to recall and social desirability bias. Third, the single-center, middle-aged sample may limit generalizability. Fourth, regarding the study design, it is important to note two considerations. Our network analysis did not employ a control group of healthy individuals or patients with other chronic conditions. This was a deliberate choice aligned with our primary aim: to model the interrelationships within the biopsychosocial system of CD, rather than to compare across populations. Additionally, participants were recruited during hospitalization, a clinical context typically associated with increased disease burden and healthcare needs. While this enhances the clinical relevance of our findings, it may limit generalizability to outpatients in remission. Should consider longitudinal or stratified designs to explore how network structures may differ across disease states (e.g., flare vs. remission). Finally, the assumption of static relationships in network analysis may not fully capture the temporal variability of CD activity and psychosocial states.

## 5 Conclusion

This study highlights self-worth, autonomy, and perceived social support as central psychosocial factors influencing disease activity in Crohn’s disease. These variables acted as key nodes and bridges within the psychosocial–clinical network. Crucially, this system is moderated by marital status. For single patients, family support and self-worth form a direct pathway to clinical severity. For married patients, friend support and autonomy are more central to adjustment, with a buffered link to disease. These findings call for context-aware personalized care. Psychosocial assessment and intervention should account for relationship status, targeting key nodes—such as family functioning for single patients or spousal and peer networks for married patients—to develop more precise biopsychosocial strategies in CD management.

## Data Availability

The raw data supporting the conclusions of this article will be made available by the authors, without undue reservation.

## References

[ref1] Barberio B. Zamani M. Black C. J. Savarino E. V. Ford A. C. (2021). Prevalence of symptoms of anxiety and depression in patients with inflammatory bowel disease: a systematic review and meta-analysis. Lancet Gastroenterol. Hepatol. 6, 359–370. doi: 10.1016/S2468-1253(21)00014-5, 33721557

[ref2] Borysenko J. Z. (1982). Behavioral--physiological factors in the development and management of cancer. Gen. Hosp. Psychiatry 4, 69–74. doi: 10.1016/0163-8343(82)90029-9, 7042459

[ref3] Calderón C. Ferrando P. J. Lorenzo-Seva U. Gómez-Sánchez D. Fernández-Montes A. Palacín-Lois M. . (2021). Multidimensional scale of perceived social support (MSPSS) in cancer patients: psychometric properties and measurement invariance. Psicothema 33, 131–138. doi: 10.7334/psicothema2020.263, 33453746

[ref4] Chabowski M. Polański J. Jankowska-Polanska B. Lomper K. Janczak D. Rosinczuk J. (2017). The acceptance of illness, the intensity of pain and the quality of life in patients with lung cancer. J. Thorac. Dis. 9, 2952–2958. doi: 10.21037/jtd.2017.08.70, 29221267 PMC5708453

[ref5] Cheifetz A. S. (2013). Management of active Crohn disease. JAMA 309, 2150–2158. doi: 10.1001/jama.2013.4466, 23695484 PMC5877483

[ref6] Chen R. B. (2016). Self-management for patients with inflammatory bowel disease in a gastroenterology ward in China: a best practice implementation project. JBI Database System Rev. Implement. Rep. 14, 271–277. doi: 10.11124/JBISRIR-2016-003180, 27941520

[ref7] Chen J. Chen Z. (2008). Extended Bayesian information criteria for model selection with large model spaces. Biometrika 95, 759–771. doi: 10.1093/biomet/asn034

[ref8] Chen J. Geng J. Wang J. Wu Z. Fu T. Sun Y. . (2022). Associations between inflammatory bowel disease, social isolation, and mortality: evidence from a longitudinal cohort study. Ther. Adv. Gastroenterol. 15:17562848221127474. doi: 10.1177/17562848221127474 PMC9528002 36199290

[ref9] Chen D. Zhang H. Shao J. Tang L. Cui N. Wang X. . (2023). Determinants of adherence to diet and exercise behaviours among individuals with metabolic syndrome based on the capability, opportunity, motivation, and behaviour model: a cross-sectional study. Eur. J. Cardiovasc. Nurs. 22, 193–200. doi: 10.1093/eurjcn/zvac034, 35672276

[ref10] Dahlhamer J. M. Zammitti E. P. Ward B. W. Wheaton A. G. Croft J. B. (2016). Prevalence of inflammatory bowel disease among adults aged ≥18 years - United States, 2015. MMWR Morb. Mortal Wkly. Rep. 65, 1166–1169. doi: 10.15585/mmwr.mm6542a3, 27787492

[ref11] de Dios-Duarte M. J. Arias A. Barrón A. (2024). Impact of psychosocial factors on the activity of Crohn's disease: a cross-sectional analysis of social support, stress, and flare-up incidence. J. Clin. Med. 13:3086. doi: 10.3390/jcm13113086, 38892797 PMC11172725

[ref12] Ding S. S. Liu C. Zhang Y. F. Sun L. P. Xiang L. H. Liu H. . (2022). Contrast-enhanced ultrasound in the assessment of Crohn's disease activity: comparison with computed tomography enterography. Radiol. Med. 127, 1068–1078. doi: 10.1007/s11547-022-01535-z, 35943658

[ref13] Epskamp S. Borsboom D. Fried E. I. (2018). Estimating psychological networks and their accuracy: a tutorial paper. Behav. Res. Methods 50, 195–212. doi: 10.3758/s13428-017-0862-1, 28342071 PMC5809547

[ref14] Felton B. J. Revenson T. A. (1984). Coping with chronic illness: a study of illness controllability and the influence of coping strategies on psychological adjustment. J. Consult. Clin. Psychol. 52, 343–353. doi: 10.1037/0022-006X.52.3.343, 6747054

[ref15] Fruchterman T. M. Reingold E. M. (1991). Graph drawing by force-directed placement. Softw. Pract. Experience 21, 1129–1164. doi: 10.1002/spe.4380211102

[ref16] Gao Z. Li S. Xu Y. Zhang M. Wang J. Bai X. (2025). Health-promoting lifestyle and associated factors among head and neck Cancer patients in Northeast China: a cross-sectional study. J. Adv. Nurs. doi: 10.1111/jan.17102 [E-pub ahead of print], 40462455

[ref17] Ghia J. E. Blennerhassett P. Deng Y. Verdu E. F. Khan W. I. Collins S. M. (2009). Reactivation of inflammatory bowel disease in a mouse model of depression. Gastroenterology 136, e2281–e2284. doi: 10.1053/j.gastro.2009.02.069 19272381

[ref18] Horrigan J. M. Louis E. Spinelli A. Travis S. Moum B. Salwen-Deremer J. . (2023). The real-world global use of patient-reported outcomes for the Care of Patients with Inflammatory Bowel Disease. Crohns Colitis 360 5:otad006. doi: 10.1093/crocol/otad006 36937140 PMC10022710

[ref19] Hu Y. Guo X. You H. Liu L. Wang Y. (2025). Mediating effect of social support on the relationships between caregiver burden and quality of life in family caregivers of people with dementia: a cross-sectional study in rural China. BMC Nurs. 24:37. doi: 10.1186/s12912-024-02671-9 39794759 PMC11721535

[ref20] Huang J. Ding S. Xiong S. Liu Z. (2021). Medication adherence and associated factors in patients with type 2 diabetes: a structural equation model. Front. Public Health 9:730845. doi: 10.3389/fpubh.2021.730845, 34805063 PMC8599446

[ref21] Huang M. Tu L. Wu L. Zou Y. Li X. Yue X. . (2023). Is disease activity associated with social support and psychological distress in Crohn's disease patients? Results of a cross-sectional study in a Chinese hospital population. BMJ Open 13:e076219. doi: 10.1136/bmjopen-2023-076219, 37879697 PMC10603502

[ref22] Jeong S. Kim H. Lho S. K. Hwang I. Mun S. Kim S. . (2025). Schema-informed digital mental health intervention for maladaptive cognitive-emotional patterns: a randomized controlled trial. J. Med. Internet Res. 27:e65892. doi: 10.2196/65892, 40574385 PMC12395104

[ref23] Kamp K. J. West P. Holmstrom A. Luo Z. Wyatt G. Given B. (2019). Systematic review of social support on psychological symptoms and self-management Behaviors among adults with inflammatory bowel disease. J. Nurs. Scholarsh. 51, 380–389. doi: 10.1111/jnu.12487, 31119856

[ref24] Kang B. Li Y. Zhao X. Cui X. Qin X. Fang S. . (2024). Negative parenting style and depression in adolescents: a moderated mediation of self-esteem and perceived social support. J. Affect. Disord. 345, 149–156. doi: 10.1016/j.jad.2023.10.132, 37879412

[ref25] Keefer L. Kiebles J. L. Taft T. H. (2011). The role of self-efficacy in inflammatory bowel disease management: preliminary validation of a disease-specific measure. Inflamm. Bowel Dis. 17, 614–620. doi: 10.1002/ibd.21314, 20848516 PMC3005084

[ref26] Larsen E. N. Brünnich Sloth M. M. Nielsen J. Osler M. Jørgensen T. S. H. (2023). The Association of Children and Their Educational Attainment with Diabetes-related Complications and mortality among older adults with type 2 diabetes: a Nationwide cohort study. Can. J. Diabetes 47, 649–657.e646. doi: 10.1016/j.jcjd.2023.07.004, 37460085

[ref27] Li X. P. Wang Y. Y. Sun Y. S. Zhang L. J. Zhao X. Y. Liu Z. Q. . (2023). Preoperative and postoperative clinical signatures of postgastrectomy venous thromboembolism in patients with gastric cancer: a retrospective cohort study. Asian J. Surg. 46, 1556–1563. doi: 10.1016/j.asjsur.2022.08.083, 36089437

[ref28] Li F. Xiao T. Qiu X. Liu C. Ma Q. Yu D. . (2025a). Oral frailty and its influencing factors in patients with cancer undergoing chemotherapy: a cross-sectional study. BMC Oral Health 25:426. doi: 10.1186/s12903-025-05789-7 40128687 PMC11934545

[ref29] Li F. Xiao T. Tang A. Wang Z. Liu C. Ma Q. . (2025b). Acceptance of illness and its relationship with benefit finding among patients with colorectal cancer undergoing chemotherapy: a latent profile analysis. Asia Pac. J. Oncol. Nurs. 12:100715. doi: 10.1016/j.apjon.2025.100715, 40485987 PMC12143793

[ref30] Lin R. Chen H. Shu W. Sun M. Fang L. Shi Y. . (2018). Clinical significance of soluble immunoglobulins A and G and their coated bacteria in feces of patients with inflammatory bowel disease. J. Transl. Med. 16:359. doi: 10.1186/s12967-018-1723-0 30558634 PMC6296095

[ref31] Liu S. Fan X. Jiang L. Liu T. (2025). Factors influencing nutritional literacy among rural older adults: a cross-sectional survey based on the theory of planned behavior. Front. Nutr. 12:1578836. doi: 10.3389/fnut.2025.1578836 40693206 PMC12277256

[ref32] Liu J. Guo M. Zhang D. Cheng S. Y. Liu M. Ding J. . (2012). Adiponectin is critical in determining susceptibility to depressive behaviors and has antidepressant-like activity. Proc. Natl. Acad. Sci. USA 109, 12248–12253. doi: 10.1073/pnas.1202835109, 22778410 PMC3409774

[ref33] Liu X. Zhang Q. Huang L. Zhuang Y. (2025). Associations between disease acceptance and dietary adherence in patients with type 2 diabetes mellitus in China: a cross-sectional study. Diabetes Res. Clin. Pract. 224:112196. doi: 10.1016/j.diabres.2025.112196, 40268141

[ref34] Ma N. Jia R. Teng Y. Fu Y. Yan X. (2025). Associations between acceptance of illness, psychological resilience, and patient activation among young and middle-aged patients with lung cancer. Patient Educ. Couns. 137:108821. doi: 10.1016/j.pec.2025.108821, 40367550

[ref35] Opheim R. Moum B. Grimstad B. T. Jahnsen J. Prytz Berset I. Hovde Ø. . (2020). Self-esteem in patients with inflammatory bowel disease. Qual. Life Res. 29, 1839–1846. doi: 10.1007/s11136-020-02467-9, 32144613 PMC7295843

[ref36] Robinaugh D. J. Millner A. J. McNally R. J. (2016). Identifying highly influential nodes in the complicated grief network. J. Abnorm. Psychol. 125, 747–757. doi: 10.1037/abn0000181, 27505622 PMC5060093

[ref37] Samuel T. Nigussie K. Mirkena Y. Azale T. (2022). Relationship between social support and schizophrenia relapse among patients with schizophrenia on follow-up at Amanuel mental specialized hospital, Addis Ababa, Ethiopia: a case-control study. Front. Psychol. 13:980614. doi: 10.3389/fpsyt.2022.980614, 36506425 PMC9730020

[ref38] Sofia M. A. Lipowska A. M. Zmeter N. Perez E. Kavitt R. Rubin D. T. (2020). Poor sleep quality in Crohn's disease is associated with disease activity and risk for hospitalization or surgery. Inflamm. Bowel Dis. 26, 1251–1259. doi: 10.1093/ibd/izz258, 31820780 PMC7365809

[ref39] Tzanetakos C. Vakouftsi V.-R. Mavridoglou G. Psarra M. Gourzoulidis G. (2025). Disease burden and unmet medical need in patients with Crohn’s disease in Greece: a cross-sectional patient survey. Ann. Gastroenterol. 38, 629–640. doi: 10.20524/aog.2025.1013 PMC12371981 40843739

[ref40] van den Brink G. Stapersma L. Vlug L. E. Rizopolous D. Bodelier A. G. van Wering H. . (2018). Clinical disease activity is associated with anxiety and depressive symptoms in adolescents and young adults with inflammatory bowel disease. Aliment. Pharmacol. Ther. 48, 358–369. doi: 10.1111/apt.14832, 29897134

[ref41] Wang C. Sheng Y. Yu L. Tian F. Xue Y. Zhai Q. (2023). Effects of cognitive behavioral therapy on mental health and quality of life in inflammatory bowel disease patients: a meta-analysis of randomized controlled trials. Behav. Brain Res. 454:114653. doi: 10.1016/j.bbr.2023.114653, 37657513

[ref42] Wu Y. C. Xiao Z. B. Lin X. H. Zheng X. Y. Cao D. R. Zhang Z. S. (2020). Dynamic contrast-enhanced magnetic resonance imaging and diffusion-weighted imaging in the activity staging of terminal ileum Crohn's disease. World J. Gastroenterol. 26, 6057–6073. doi: 10.3748/wjg.v26.i39.6057, 33132655 PMC7584052

[ref43] Wu Q. Zhu P. Liu X. Chen C. Ji Q. Gu Q. (2022). The impact of family function on mental health status in patient with inflammatory bowel disease: the mediating role of self-esteem. Front. Psychol. 13:1007318. doi: 10.3389/fpsyt.2022.1007318 PMC9754665 36532185

[ref44] Xiong Q. Tang F. Li Y. Xie F. Yuan L. Yao C. . (2022). Association of inflammatory bowel disease with suicidal ideation, suicide attempts, and suicide: a systematic review and meta-analysis. J. Psychosom. Res. 160:110983. doi: 10.1016/j.jpsychores.2022.110983, 35872532

[ref45] Zhao L. Sun Q. Guo Y. Yan R. Lv Y. (2022). Mediation effect of perceived social support and resilience between physical disability and depression in acute stroke patients in China: a cross-sectional survey. J. Affect. Disord. 308, 155–159. doi: 10.1016/j.jad.2022.04.034, 35429523

[ref46] Zhou K. Huang X. Chen M. Li Z. Qin J. Ji Y. . (2024). Pre-hospital symptom clusters and symptom network analysis in decompensated cirrhotic patients: a cross-sectional study. J. Adv. Nurs. 80, 2785–2800. doi: 10.1111/jan.16044, 38197541

[ref47] Zimet G. D. Powell S. S. Farley G. K. Werkman S. Berkoff K. A. (1990). Psychometric characteristics of the multidimensional scale of perceived social support. J. Pers. Assess. 55, 610–617. doi: 10.1080/00223891.1990.9674095, 2280326

